# A Review of the Prognostic Significance of Neutrophil-to-Lymphocyte Ratio in Nonhematologic Malignancies

**DOI:** 10.3390/diagnostics14182057

**Published:** 2024-09-16

**Authors:** Defne Cigdem Koc, Ion Bogdan Mănescu, Măriuca Mănescu, Minodora Dobreanu

**Affiliations:** 1 Medical Campus Hamburg, George Emil Palade University of Medicine, Pharmacy, Science, and Technology of Targu Mures, 11-15 Albert-Einstein-Ring, 22761 Hamburg, Germany; defne.koc@live.de (D.C.K.); bogdan.manescu@umfst.ro (I.B.M.); 2 Department of Laboratory Medicine, Faculty of Medicine, George Emil Palade University of Medicine, Pharmacy, Science, and Technology of Targu Mures, 38 Gheorghe Marinescu, 540142 Targu Mures, Romania; 3 Department of Pediatrics, Emergency County Clinical Hospital of Targu Mures, 50 Gheorghe Marinescu, 540136 Targu Mures, Romania; 4 Clinical Laboratory, Emergency County Clinical Hospital of Targu Mures, 50 Gheorghe Marinescu, 540136 Targu Mures, Romania

**Keywords:** cancer, clinical outcome, neutrophil-to-lymphocyte ratio, NLR, nonhematologic malignancy, prognosis

## Abstract

Biomarkers are crucial in cancer diagnostics, prognosis, and surveillance. Extensive research has been dedicated to identifying biomarkers that are broadly applicable across multiple cancer types and can be easily obtained from routine investigations such as blood cell counts. One such biomarker, the neutrophil-to-lymphocyte ratio (NLR), has been established as a prognostic marker in cancer. However, due to the dynamic nature of cancer diagnosis and treatment, periodic updates are necessary to keep abreast of the vast amount of published data. In this review, we searched the PubMed database and analyzed and synthesized recent literature (2018–February 2024) on the role of NLR in predicting clinical outcomes in nonhematologic malignancies. The search was conducted using the PubMed database. We included a total of 88 studies, encompassing 28,050 human subjects, and categorized the findings into four major groups: gastrointestinal cancer, cancers of the urinary tract and reproductive system, lung cancer, and breast cancer. Our analysis confirms that NLR is a reliable prognostic indicator in cancer, and we discuss the specific characteristics, limitations, and exceptions associated with its use. The review concludes with a concise Q&A section, presenting the most relevant take-home messages in response to five key practical questions on this topic.

## 1. Introduction

Biomarkers play a pivotal role in cancer diagnostics, prognosis and prediction. While most clinically used cancer biomarkers correspond to specific biomolecules such as proteins or nucleic acids, they are often limited to certain cancer types. Research has been conducted to identify biomarkers that are not only widely applicable to several cancer types but can also be obtained easily from routine investigations such as blood cell counts. One such biomarker is the neutrophil-to-lymphocyte ratio (NLR), which is obtained by dividing the absolute neutrophil count by the absolute lymphocyte count.

Composed of two highly dynamic blood cell counts, NLR can change rapidly in response to inflammation. In cancer, the tumor microenvironment is marked by chronic inflammation, with leukocytes impacting tumor growth and progression [1,2,3]. Neutrophils, for instance, may play pro- or anti-tumorigenic roles, depending on the context [2,3]. However, neutrophils often promote a pro-tumoral environment, favoring metastasis through several mechanisms [2,3]. These include the release of reactive oxygen species that induce genomic instability, the secretion of proteases, and growth factors that facilitate cancer cell invasion and metastasis, promoting tumor angiogenesis, and impeding the adaptive immune response against the tumor [1,2,3]. Conversely, lymphocytes, particularly tumor-infiltrating T lymphocytes, exhibit anti-tumoral activities in certain cancer types, attacking tumor cells and potentially controlling tumor growth [1,4,5,6]. The balance between these pro-tumoral and anti-tumoral forces is reflected by the NLR, which highlights its role in assessing cancer prognosis. Thus, an increased NLR is often associated with unfavorable outcomes in several, particularly solid, malignancies [1,7,8,9]. The NLR, by reflecting key inflammatory processes within the tumor microenvironment, offers not only insights into the current state of disease but also serves as a bridge to more tailored therapeutic strategies. In the evolving landscape of precision medicine, such insights are critical. They allow clinicians to select treatments that are most likely to be effective and detect therapeutic failure earlier, facilitating a more targeted approach to cancer management. This capability to adapt to the individual needs of each patient is what positions NLR as a promising tool in modern oncology.

This review will examine the value of NLR in predicting clinical outcomes in patients with nonhematologic malignancies, with a focus on key oncological endpoints such as overall survival (OS) and progression-free survival (PFS). In addressing this objective, we have considered all relevant statistical measures of predictive performance, although the majority of studies utilize odds ratios (OR) and hazard ratios (HR). The role of NLR in cancer risk prediction or as a diagnostic tool for initial cancer detection falls outside the scope of this review.

## 2. Materials and Methods

### 2.1. Database Search

The electronic PubMed database was explored on 26 February 2024 to identify recent and relevant articles regarding the predictive value of NLR in cancer. To optimize search results, the following search query was used: ((NLR) OR (neutrophil-to-lymphocyte ratio) OR (neutrophil to lymphocyte ratio) OR (neutrophil-lymphocyte ratio) OR (neutrophil lymphocyte ratio)) AND ((progno*) OR (prediction) OR (predictive) OR (outcome)) AND ((marker) OR (biomarker)) AND ((cancer) OR (carcinoma) OR (malignancy) OR (malignant)). After filtering results by year of publication (2018–2024), article type (original research only), species (human), and language (English), the database search yielded a total of 245 articles.

### 2.2. Exclusion Criteria and Included Articles

**Figure 1:**
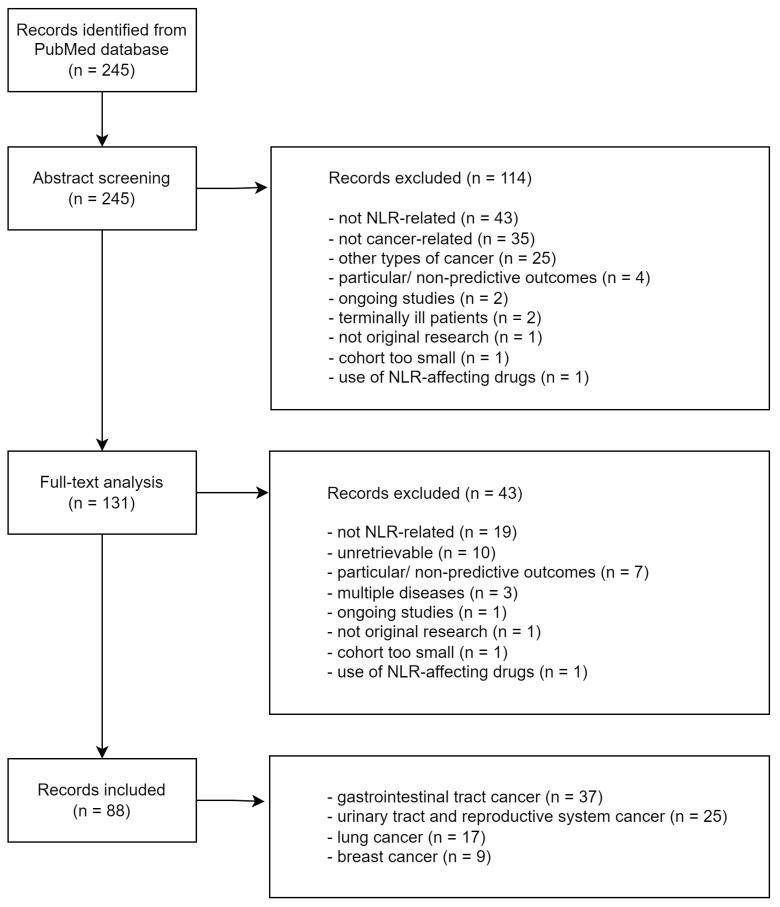
Article selection flowchart.

Following abstract screening by two independent researchers, a total of 114 articles were excluded from the review. Among these, 25 articles initially met the general inclusion criteria but were subsequently excluded due to the diversity of cancer types studied, which precluded their categorization into distinct groups. After full-text analysis of the remaining 131 articles, the same two researchers further excluded 43 articles. Thus, the present review includes 88 selected articles divided into four main groups: cancers of the urinary tract and reproductive system (*n* = 25), lung cancer (*n* = 17), breast cancer (*n* = 9), and cancers of the gastrointestinal tract (*n* = 37). Exclusion criteria comprised: not peer reviewed, not NLR-related (including studies where NLR was part of a predictive score), not cancer-related, particular/non-predictive reported outcomes, ongoing trial, terminally ill patients, cohort too small (*n* < 20), not original research (except for post hoc analyses), studies involving NLR-affecting drugs (other than cancer treatment), multiple diseases (multiple types of cancer or cancer with other concurrent diseases), and data of interest unavailable (full text unretrievable with insufficient information in the abstract). Details on excluded articles in each step of the analysis are provided in the article selection flowchart (Figure 1). During the selection process, no additional quality criteria, such as risk of bias assessments, were applied. No meta-analysis of the referenced articles was conducted.

While the primary objective of this review did not involve an extensive evaluation of the pathophysiological mechanisms underlying the predictive significance of NLR in cancer, or its variation during/after cancer treatment, relevant studies beyond those encompassed within the review were referenced for elucidation, clarification, or contextualization purposes. To aid in summarizing the content, a brief conclusion and a table outlining the key aspects of relevant studies are provided at the end of each section (Table 1, Table 2, Table 3, Table 4, Table 5, Table 6 and Table 7). However, some studies are referenced solely within the main text. For numerical data not explicitly mentioned (e.g., cohort size, NLR cut-off values, hazard ratios, etc.), readers can refer to the tables at the end of each section.

## 3. Results

### 3.1. Cancers of the Gastrointestinal Tract

Gastrointestinal cancers constitute over a quarter of all cancers [10,11]. With increasing prevalence, gastrointestinal cancers rank first in terms of incidence and mortality [10]. Colorectal cancer, which is the third most diagnosed malignancy and the second leading cause of cancer-related death, has seen an alarming increase in young adults over the past few decades [10,11]. Overall, gastrointestinal cancers pose a significant public health concern with far-reaching socioeconomic implications [10,11].

#### 3.1.1. Gastric and Esophageal Cancer

The eleven studies included in this section have explored the prognostic value of the NLR in gastro-esophageal cancer. Among these, three distinct retrospective studies on advanced gastric cancer underlined statistically significant associations between elevated NLR and decreased overall survival (OS) or progression-free survival (PFS) [12,13,14]. In a study including 61 patients with gastric and gastro-esophageal cancer, Magdy et al. further validated earlier-mentioned findings by demonstrating an association between pretreatment NLR and both event-free survival (EFS) and OS [15]. Similar findings, along with a dynamic prognostic potential of the NLR, were recorded in metastatic cancer as well. In a cohort of 116 patients, not only did a baseline NLR of 3.95 or higher significantly predict poorer OS (*p* = 0.003), but an increase in NLR during chemotherapy, referred to as c-NLR (change in NLR), was also correlated with decreased survival rates (*p* = 0.003) [16].

Apart from predicting major clinical outcomes, the NLR shows potential use as a biomarker for the prediction of treatment efficacy in gastric cancer. In a multicenter retrospective study, Nakazawa et al. noted that a high pre-treatment (*p* = 0.045) and post-treatment NLR (*p* = 0.025) after two courses of nivolumab correlated with therapeutic resistance [17]. Furthermore, Kim et al. demonstrated that a lower NLR was generally linked with higher PFS and a more favorable response in patients receiving nivolumab [14]. Conversely, a study on 55 advanced esophageal cancer patients on pembrolizumab-based regimens reported that the NLR did not predict the efficacy of immune-checkpoint-inhibitors (*p* = 0.457) [18].

In addition to the NLR’s predictive role in immunotherapy, several studies assessed its relevance as a marker in surgically treated patients. Sato et al. conducted a retrospective cohort study of 121 gastric cancer patients undergoing curative resection where a high post-surgical NLR was found to be an independent prognostic factor for worse OS (*p* = 0.027) [19]. In contrast, several studies with different clinical settings revealed either negative or inconclusive results regarding the association between NLR and clinical outcomes: Wen et al. (esophagectomy/gastrectomy, *p* = 0.210) [20], Pan et al. (resectable gastric cancer) [21], and Li et al. (gastric cancer treated by neoadjuvant chemotherapy and D2 lymphadenectomy, *p* = 0.059) [22].

**Table 1:** The predictive value of neutrophil-to-lymphocyte ratio (NLR) as reported by studies on gastro-esophageal cancer.

Cancer Type	Study Details/Treatment	*N*	Median Follow-Up	Outcome	NLR Predictor/ Cut-Off	Risk [95% CI]/ Significance	Ref.
HER2+ aGC	First-line trastuzumab treatment	112	-	OS (poorer)	Higher	*p* = 0.002	[12]
				PFS (poorer)		*p* = 0.005	
aGC	Treated with chemotherapy	216	-	OS (poorer)	≥3.06	HR 1.61 [1.18–2.21]	[13]
aGC	Nivolumab monotherapy after failure of ≥2 lines of chemotherapy	45	28.3 mo	OS (better)	≤2.90	HR 0.34 [0.17–0.70]	[14]
				PFS (better)		HR 0.56 [0.30–1.04]	
GC and GEC	All cancer stages, no prior treatment	61	8.0 mo	OS (poorer)	>2.40	*p* = 0.013	[15]
				EFS (poorer)		*p* = 0.001	
mGC	Baseline and after 2 first-line chemotherapy cycles	116	8.7 mo	OS (poorer)	≥3.96 (baseline)	HR 2.16 [1.29–3.61]	[16]
					Increase after treatment	HR 2.36 [1.35–4.13]	
Postop. recurrent or unresectable aGC	Nivolumab treatment	58	-	Disease progression/low sensitivity to nivolumab	≥5.00 (pretreatment)	*p* = 0.045	[17]
					≥5.00 (after treatment)	*p* = 0.025	
Advanced ESCC	Chemoradiotherapy + pembrolizumab	55	24.0 mo	OS (poorer)	≥2.43	HR 1.51 [0.49–4.63]	[18]
GC	Macroscopically curative resection	121	-	OS (poorer)	>3.00 (after resection)	HR 1.50 [1.05–2.16]	[19]
GC or (G) EC	Gastrectomy or esophagectomy	723	-	OS	≥5.00	HR 1.20 [0.90–1.61]	[20]
GC	Tumorectomy + D2 lymphadenectomy	870	59.9 mo	OS	>1.44	*p* > 0.05	[21]
GC	Gastrectomy + ≥2 cycles of NACT	225	29.8 mo	OS (poorer)	Higher (pre-NACT)	HR 1.17 [1.00–1.35]	[22]

Abbreviations: CI confidence interval, (G) EC (gastro-) esophageal cancer, EFS event-free survival, ESCC esophageal squamous cell carcinoma, (a/m) GC (advanced/metastatic) gastric cancer, GEC gastro-esophageal cancer, HER2+ human epidermal growth factor receptor 2 positive, HR hazard ratio, mo months, NACT neoadjuvant chemotherapy, OS overall survival, PFS progression-free survival, Ref reference.

Overall, the NLR was identified as a general prognostic marker in gastric cancer, demonstrating predictive value in immunotherapy outcomes. However, its benefit in the context of surgical treatment is inconsistent and no clear benefit could be observed in neoadjuvant chemotherapy combined with lymphadenectomy. Similarly, the predictive significance of the NLR in esophageal cancer treatment is inconclusive and yet to be determined by further studies. This uncertainty should be interpreted considering the limited number of studies on esophageal malignancies included in this review.

#### 3.1.2. Pancreatic Cancer

Six studies included in this section have explored the prognostic value of the NLR in pancreatic (ductal) adenocarcinoma, which is the most common type of pancreatic cancer. Among these, two studies reported on surgically-treated pancreatic cancer. Schlanger et al. found that, in patients eligible for curative surgical resection, neither preoperative (*p* = 0.243), nor postoperative (*p* = 0.373) NLR values were statistically significant for overall survival [23]. Similar findings were reported by Xu et al. in a retrospective study of patients with indication for surgical treatment and no prior radiotherapy and/or neoadjuvant chemotherapy (*p* = 0.892 for OS) [24].

On the contrary, the NLR seems to be prognostic for clinical outcomes in more advanced forms of pancreatic cancer. Vasconcelos de Matos et al. reported a significant (*p* < 0.001) association between the NLR and survival in patients with advanced pancreatic cancer treated with chemotherapy [25]. In a smaller study of advanced pancreatic cancer patients treated with a combination of agonistic anti-CD40 monoclonal antibodies and chemotherapy (gemcitabine), Wattenberg et al. reported significantly worse OS in patients with high NLR (*p* = 0.043), suggesting a possible resistance to treatment in states of systemic inflammation [26]. Similar findings were reported by Mc Lellan et al. in a larger study, where NLR was an independent prognostic factor for OS (*p* = 0.001) and PFS (*p* = 0.0026) in metastatic pancreatic cancer patients treated with chemotherapy [27]. Additionally, a significant dynamic role was observed over time, showing an association between poor treatment response and higher median NLR, consequently suggesting a poor prognosis [27]. Nevertheless, these findings are challenged by a larger post hoc analysis of two clinical trials on metastatic pancreatic cancer, which failed to show a statistically significant independent prognostic value of the NLR for OS (*p* = 0.70) [28].

**Table 2:** The predictive value of neutrophil-to-lymphocyte ratio (NLR) as reported by studies on pancreatic (ductal) adenocarcinoma.

Cancer Type	Study Details/Treatment	*N*	Median Follow-Up	Outcome	NLR Predictor/ Cut-Off	Risk [95% CI]/ Significance	Ref.
PDAC	Curative surgical resection	312	-	OS	Higher (preoperative)	OR 1.20 [0.90–1.80] *	[23]
					Higher (postoperative)	OR 1.20 [0.82–1.70] *	
PDAC	Surgical treatment	148	12.0 mo	OS	>2.04	HR 1.02 [0.72–1.45]	[24]
aPDAC	Chemotherapy	65	14.8 mo	OS (poorer)	>5.00	HR 6.20 [2.59–14.87]	[25]
aPDAC	Anti-CD40 + gemcitabine	22	-	OS (poorer)	>3.10	HR 3.87 [1.04–14.38]	[26]
mPDAC	Chemotherapy	212	-	OS (poorer)	>5.00	HR 2.01 [1.33–3.05]	[27]
				PFS (poorer)		HR 1.80 [1.23–2.65]	
mPDAC	Post hoc analysis of two clinical trials	372	-	OS	≥3.20	HR 1.08 [0.73–1.60]	[28]

Abbreviations: CI confidence interval, HR hazard ratio, mo months, OS overall survival, (a/m) PDAC (advanced/metastatic) pancreatic ductal adenocarcinoma, PFS progression-free survival, Ref reference. * Univariate analysis.

Overall, the NLR seems to be a prognostic factor for clinical outcomes in patients with more advanced forms of pancreatic cancer who are treated with chemo- and/or immunotherapy, rather than in patients with early stages of pancreatic cancer which only have indication for surgical treatment. This observation mirrors the one found in the gastro-esophageal cancer section.

#### 3.1.3. Cancers of the Liver and Biliary System

Four studies included in this section have explored the prognostic value of the NLR in hepatocellular carcinoma (HCC). While using different NLR cut-off values, Zhou et al. (NLR > 1.79) as well as Deng et al. (NLR > 2.31) reported significant associations between the preoperative NLR and OS (both *p* < 0.001) in two similarly sized cohorts of patients with resectable HCC [29,30]. Compared with other articles included in the present review, the cut-off value used by Zhou et al. was lower, but elevated NLR values were also associated with poor differentiation observed during pathological examination [29]. The association of increased NLR (≥ 3.45) with poorer OS was also reported by the OncoBridge Study Group in a multicentric retrospective study that included HCC patients in various TNM stages from Turkey, Russia, Georgia, and Greece (*p* < 0.05) [31]. Furthermore, the potential of the NLR predicting clinical outcomes was shown in a smaller cohort of unresectable HCC patients treated with a triple therapy consisting of lenvatinib, programmed death-1 inhibitors, and transcatheter arterial chemoembolization [32]. In this prospective observational study, Qu et al. reported that NLR < 3.2 was associated with longer OS, PFS, and early tumor shrinkage (*p* < 0.001 for all), indicating a role of the NLR for optimized patient selection [32]. As for cholangiocarcinoma, only one study was included in the present review: a large retrospective international analysis of patients undergoing surgical resection for intrahepatic cholangiocarcinoma, where NLR > 5 was reported to be an independent prognostic marker for worse overall survival [33].

Regarding gallbladder cancer, only three studies were included in the current review. Cui et al. reported that NLR ≥ 4.39 was associated with shorter OS (*p* = 0.01) in a mixed Chinese cohort of patients with gallbladder cancer of various TNM stages that had received various treatments [34]. These findings were supported by another Chinese study using a similar NLR cut-off (4.33) for the prediction of OS (*p* < 0.001) in patients with gallbladder cancer undergoing curative-intent resection [35]. In a larger, multi-institutional cohort, Cotter et al. determined that OS was also predicted by the preoperative NLR in patients with gallbladder cancer undergoing curative-intent resection, but only in association with other factors [36]. With the help of machine learning, patients were classified in groups, with the highest OS (*p* < 0.0001) being observed in the group meeting the following criteria: NLR ≤1.5, tumor size < 5 cm, low CA19-9 levels, and no preoperative biliary drainage [36]. Although this study did not assess an independent prognostic role for NLR, it highlights NLR’s potential in risk stratification and guiding curative surgery decisions [36].

**Table 3:** The predictive value of neutrophil-to-lymphocyte ratio (NLR) as reported by studies on cancers of the liver and biliary system.

Cancer Type	Study Details/Treatment	*N*	Median Follow-Up	Outcome	NLR Predictor/Cut-Off	Risk [95% CI]/ Significance	Ref.
Nonmetastatic HCC	Surgical treatment: liver resection	136	>38.0 mo	OS (poorer)	>1.79	RR 3.14 [2.36–4.17]	[29]
HCC	Surgical treatment: radical resection	150	-	OS (poorer)	>2.31	HR 2.50 [1.45–4.30]	[30]
HCC	Various TNM stages and treatments	630	-	OS (poorer)	≥3.45	HR 2.02 [1.01–4.13]	[31]
Unresectable HCC	Triple therapy: lenvatinib, PD-1 inhibitors, and transcatheter arterial chemoembolization	63	20.8 mo	OS (better)	<3.2	HR 0.14 [0.06–0.35]	[32]
				PFS (better)		*p* < 0.001	
				Early tumor shrinkage		*p* < 0.001	
Intrahepatic cholangiocarcinoma	Surgical resection	688	22.3 mo	OS (poorer)	>5.00	HR 1.58 [1.10–2.27]	[33]
Gallbladder cancer	Various TNM stages and treatments	159	8.0 mo	OS (poorer)	≥4.39	HR 1.57 [1.11–2.22]	[34]
Gallbladder cancer	Curative-intent resection	90	-	OS (poorer)	≥4.33	HR 3.84 [2.12–6.94]	[35]

Abbreviations: CI confidence interval, HCC hepatocellular carcinoma, HR hazard ratio, mo months, OS overall survival, PD-1 programmed death-1, PFS progression-free survival, Ref reference, RR relative risk.

Overall, despite the limited number of recent studies included in this review, evidence suggests that the NLR can be used as a predictive tool for cancers of the liver and biliary system, whether the cancer is resectable or more advanced.

#### 3.1.4. Colorectal Cancer

Twelve studies included in this section have explored the prognostic value of the NLR in colorectal cancer (CRC). Among these, two focused broadly on colorectal cancer patients, eight investigated metastatic colorectal cancer, and the remaining two targeted rectal cancer specifically.

In a retrospective analysis of a mixed cohort of CRC patients in various TNM stages, with or without metastases, Silva et al. found that NLR ≥ 3.0 was an independent poor prognostic factor for OS (*p* = 0.001) [37]. In a prospective multicenter study of patients with nonmetastatic CRC undergoing radical colorectal surgery, a significant association between the NLR and DFS (*p* = 0.048) was shown only in colon cancer, not rectal cancer (*p* = 0.403) [38]. Additionally, no statistically significant association between NLR and OS was found, presumed to be related to the relatively short median follow-up time of 24 months [38].

As medical therapies shift towards tailored and individualized approaches, several studies have focused on exploring how certain mutations influence patient outcome and treatment response in CRC. For instance, Ciardiello et al. sought to explore the role of the NLR as a biomarker for better patient selection by conducting a post hoc analysis of the CAVE trial in metastatic CRC patients treated with cetuximab plus avelumab as rechallenge therapy [39]. Patients in the intent-to-treat group with a baseline NLR ≥ 3.0 exhibited a median overall survival (OS) that was 50% shorter compared to those with a baseline NLR < 3.0 (*p* = 0.006) [31]. Further analyses validated this association, but specifically for patients with RAS/BRAF wild-type (*p* = 0.005), not mutant ctDNA (*p* = 0.53) [39]. RAS wild-type ctDNA patients with a low NLR also showed a significantly longer survival when treated with cetuximab plus avelumab (*p* = 0.005). Although Ciardiello et al. found no NLR association in RAS/BRAF mutant ctDNA patients [39], a much larger Italian study by Loupakis et al. identified NLR ≥ 3.0 as a prognostic marker for OS within the V600EBRAF-mutated subset of metastatic CRC patients, underlining its importance in this specific genetic context [40]. This finding was supported by a smaller retrospective study by Martinez-Lago et al. which also reported that NLR ≥ 3.0 was a negative prognostic factor in metastatic CRC patients with the V600EBRAF mutation (*p* = 0.014) [41].

Building on the aforementioned studies, the NLR has been identified as a marker of clinical outcomes in metastatic CRC across various therapeutic settings. In a retrospective multicenter study of patients with metastatic CRC receiving trifluridine/tipiracil, Stavraka et al. reported that a baseline NLR < 5.0 was significantly associated with improved survival outcomes (*p* = 0.01) [42]. This association was also evident in a smaller prospective multicenter cohort, where an NLR < 4.1 predicted better PFS (*p* = 0.04) and OS (*p* = 0.003) in metastatic CRC patients treated with the angiogenesis inhibitor apatinib [43]. Furthermore, in an open-label, single-arm, multicenter prospective study of metastatic CRC patients treated with bevacizumab, Clarke et al. found a 60% higher risk of death in patients with a baseline NLR > 5.0, nearing statistical significance (*p* = 0.052). This trend toward reduced OS and PFS aligns with other findings discussed here [44].

In addition to its association with cancers driven by specific mutations, the NLR may be utilized for stratifying metastatic CRC patients for certain treatments, as evidenced by several studies mentioned above. For instance, Ciardiello et al. indicated that the NLR could be a valuable biomarker for identifying candidates likely to benefit from cetuximab plus avelumab therapy in RAS/BRAF wild-type ctDNA CRC patients [39]. Martinez-Lago et al. suggested that an elevated NLR may negatively predict survival in CRC patients with the V600EBRAF mutation undergoing first-line antiangiogenic-based treatment [41]. Stavraka et al. reported that a low NLR could predict a favorable response to trifluridine/tipiracil treatment [42], while Wang et al. indicated that a higher NLR negatively predicts the outcome of apatinib treatment in chemotherapy-refractory metastatic CRC patients [43].

While most studies in this section focused on primary CRC, we identified two that specifically address CRC metastases. Erstad et al. demonstrated an association between NLR values and survival rates in patients with resectable colorectal liver metastases [45]. In their study, an NLR ≥ 5.0 was independently linked to poorer survival outcomes (*p* = 0.032) [37]. Similarly, Weiner et al. highlighted the potential of NLR in guiding treatment decisions [46]. They found that an NLR ≥ 5 was independently associated with a higher risk of death in patients undergoing radioembolization for colorectal liver metastases (*p* = 0.0002) [46].

Despite numerous studies on colorectal cancer, the differences in tumor subtypes and stages necessitate more comprehensive and specific research. Thus, two of the selected studies focused specifically on rectal cancer. Ke et al. based their retrospective study on rectal cancer patients receiving neoadjuvant concurrent chemoradiotherapy followed by total mesorectal excision [47]. The study revealed NLR > 3.5 to be an independent predictor of worse 5-year DFS (*p* = 0.002) and OS (*p* = 0.04) [47]. In comparison, Dudani et al. studied a more specific cohort, centering their research on locally advanced (stages II–III) rectal cancer patients undergoing neoadjuvant concurrent chemoradiotherapy [48]. This multi-institutional retrospective study reviewed a much larger sample size across eight Canadian cancer centers, with a median follow-up time of approximately six years and an NLR cutoff value of 4.0, but no predictive role of NLR was recorded for DFS (*p* = 0.14) or OS (*p* = 0.99) [48]. This presumed difference between the two studies reflects the findings of the previously mentioned prospective study from Biró et al. [38].

**Table 4:** The predictive value of neutrophil-to-lymphocyte ratio (NLR) as reported by studies on colorectal cancer (CRC).

Cancer Type	Study Details/Treatment	*N*	Median Follow-Up	Outcome	NLR Predictor/Cut-Off		Risk [95% CI]/ Significance	Ref.
CRC	Various TNM stages	148	60.0 mo	OS (poorer)	≥3.00		HR 3.64 [1.70–7.77]	[37]
CRC	Nonmetastatic, radical colorectal surgery	201	24.0 mo	DFS (poorer)	Colon	>3.96	HR 1.35 [1.00–1.81]	[38]
				DFS	Rectal		HR 1.14 [0.84–1.55]	
mCRC	Intent-to-treat group	77	23.1 mo	OS (better)	<3.00		HR 0.50 [0.30–0.80]	[39]
	RAS/BRAF wild-type ctDNA patients treated with cetuximab plus avelumab						HR 0.38 [0.19–0.75]	
mCRC	V600EBRAF mutation	395	33.9 mo	OS (poorer)	≥3.00		HR 1.54 [1.22–1.97]	[40]
mCRC	V600EBRAF mutation; chemotherapy plus antiangiogenic treatment	64	69.1 mo	OS (poorer)	≥3.00		HR 1.93 [1.10–3.30]	[42]
mCRC	Trifluridine/tipiracil treatment	236	-	OS (better)	<5.00		HR 0.56 [0.35–0.88]	[42]
				Treatment response (better)			OR 3.49 [0.97–12.5]	
mCRC	Chemotherapy resistance; treatment with apatinib	48	10.3 mo	OS (better)	<4.10		HR 0.34 [0.17–0.70]	[43]
				PFS (better)			HR 0.22 [0.05–0.93]	
mCRC	Treatment with bevacizumab	128	-	OS (poorer)	>5.00		HR 1.60 [1.00–2.70]	[44]
				PFS (poorer)			HR 1.40 [0.90–2.20]	
mCRC	Resectable liver metastases	151	41.3 mo	OS (poorer)	≥5.00		HR 2.46 [1.08–5.60]	[45]
mCRC	Liver metastases + radioembolization	131	-	OS (poorer)	≥5.00		HR 2.22 [1.46–3.38]	[46]
Rectal cancer	Neoadj. concurrent chemoradiotherapy + total mesorectal excision	184	72.7 mo	5-year OS (poorer)	>3.50		HR 1.87 [1.03–3.40]	[47]
				5-year DFS (poorer)			HR 2.80 [1.47–5.41]	
Rectal cancer	Locally advanced (stages II–III); neoadj. concurrent chemoradiotherapy	1237	71.0 mo	OS	≥4.00		HR 1.00 [0.76–1.32]	[48]
				DFS			HR 1.19 [0.95–1.50]	

Abbreviations: CI confidence interval, (m)CRC (metastatic) colorectal cancer, DFS disease-free survival, HR hazard ratio, mo months, OR odds ratio, OS overall survival, PFS progression-free survival, Ref reference.

Overall, NLR appears to be a robust predictive factor for clinical outcomes in CRC, particularly in advanced metastatic forms. However, the disparate findings of Biró et al. [38] and Dudani et al. [48] suggest that the role of NLR in rectal cancer is rather subtle, as opposed to colon cancer. This highlights the need for studies with more homogeneous patient cohorts. Future analyses on the role of NLR in CRC prognosis should aim to separately evaluate rectal and colon cancers, distinguish between cancer stages, and consider the presence of metastatic disease. NLR may also play a role in precision medicine by aiding in the stratification of patients for specific therapies or within certain genetic contexts. However, this potential has yet to be mechanistically proven and clinically validated, and therefore remains to be addressed by future investigations.

### 3.2. Cancers of the Urinary Tract and Reproductive System

We selected a total of 25 studies reporting on cancers of the urinary tract and reproductive system. These studies were grouped into categories and are briefly presented below. The predictive value of NLR for clinical outcomes was summarized in Table 5, but some studies are only discussed in the text.

#### 3.2.1. Prostate Cancer

In the CARD study, higher baseline NLR was associated with reduced OS in metastatic castration-resistant prostate cancer (mCRPC) [49]. Moreover, high baseline NLR was associated with longer radiographic progression-free survival (rPFS) in patients treated with cabazitaxel compared with those treated with abiraterone/enzalutamide (8.5 months vs. 2.8 months). A similar but weaker association was found in those with low baseline NLR (7.5 months vs. 5.1 months) [49]. A similar finding regarding abiraterone acetate was reported by the COU302 study which randomized asymptomatic or minimally symptomatic mCRPC patients to receive either abiraterone acetate plus prednisone, or prednisone (placebo), and used a NLR cut-off of 2.5 [50]. Survival analysis showed that higher NLR values were associated with poorer OS and PSA response in those treated with abiraterone acetate, but not in the placebo group. Conversely, abiraterone acetate treatment was associated with higher OS than placebo in patients with lower NLR. No association was found with PFS [50].

A multicenter retrospective analysis of M0/M1 CRPC patients with pre- or post-docetaxel androgen receptor axis-targeted treatment showed that NLR ≥ 2.5 is an independent risk factor for poorer cancer-specific survival (CSS) and rPFS [51]. Among those with NLR < 2.5, the post-docetaxel group exhibited higher 1-year rPFS (*p* = 0.031) and higher 2-year CSS (*p* = 0.026). When NLR ≥ 2.5, there were no differences in rPFS and CSS between pre- and post-docetaxel groups [51].

#### 3.2.2. Renal Cell Carcinoma

Several studies have looked into the predictive value of NLR in patients with renal cell carcinoma (RCC) who were treated surgically. For instance, a large multi-institutional retrospective study of RCC patients treated with nephrectomy reported that NLR > 3.38 was found to be associated with poorer OS (HR 2.74, [2.00–3.73], *p* < 0.001) and poorer cancer-specific survival (HR 3.42, [2.38–4.88], *p* < 0.001) in univariate analysis, but not in multivariate analysis [52]. However, NLR was significantly associated with recurrence-free survival (RFS) in both uni- and multivariate analysis [52]. A similarly sized large single-center retrospective study of patients who underwent nephrectomy for nonmetastatic clear cell RCC reported that preoperative NLR > 3.3 was associated with higher cancer-specific mortality (HR 2.66, 95% CI 1.65–4.31, *p* < 0.001) and overall mortality (HR 2.35, [1.66–3.31], *p* < 0.001) also in univariate analysis, but not in multivariate analysis [53].

When advanced or metastatic (a/m), RCC is unlikely to be treated surgically, and therefore drugs are used as first-line therapy, either chemo- or immunotherapy. Thus, several studies have analyzed the predictive value of NLR in patients with advanced RCC who were given immunotherapy in the form of tyrosine kinase inhibitors (TKIs) or immune checkpoint inhibitors (ICIs), such as anti-PD-1, anti-PD-L1, or anti-CTLA-4. NLR as a prognosticator of OS was controversial in mRCC patients treated with TKIs. A retrospective study of 150 mRCC patients treated with TKIs reported that NLR >2.0 was associated with OS in univariate (HR 1.93 [1.26–2.96], *p* = 0.002), but not multivariate analysis [54]. However, a similar study of 110 mRCC patients treated with TKIs reported that NLR was associated with OS in both univariate and multivariate analysis [55]. As for immune checkpoint inhibitors, a French multicentric retrospective study looked at the variation of NLR in 86 mRCC patients and 75 metastatic non-small cell lung cancer who received anti-PD1 nivolumab monotherapy as a second line treatment or later [56]. For the whole cohort, any NLR increase at week 6 was associated with poorer PFS (*p* < 0.0001) and OS (*p* = 0.013) compared to cases where NLR decreased [56]. Multivariate analysis also showed that an increase in NLR was associated with worse PFS and OS [56]. Another study on 52 mRCC patients treated with nivolumab reported similar findings: baseline NLR was not associated with PFS or OS, but NLR ≥ 3.0 at 4 weeks after treatment initiation was shown to be a predictor of poorer 1-year OS (*p* = 0.034) and PFS (*p* = 0.013) compared with those who had an NLR < 3.0 [57]. Similar findings were reported when nivolumab was combined with another immunotherapy drug (the anti-CTLA-4 ipilimumab), although at a higher NLR cut-off (>4.6) [58]. This small multicenter retrospective study of 35 patients with advanced or metastatic RCC reported significantly higher 1-year PFS in patients with NLR ≤ 4.6 compared with those with NLR > 4.6 (*p* = 0.04) [58]. Even when immunotherapy (anti-PD-L1) is combined with another class of drugs (TKIs), the results are similar [59]. This was reported by a larger study, the first interim analysis of the phase 3 JAVELIN Renal 101 trial, which revealed that patients with aRCC treated with avelumab plus either axitinib or with sunitinib showed better PFS and OS rates when NLR was below the median value (<2.8, in dichotomous analysis) or generally lower (in multivariate continuous analysis), especially for the avelumab plus sunitinib arm [59].

#### 3.2.3. Urothelial and Bladder Cancer

A large multi-center retrospective study of patients with localized upper tract urothelial carcinoma (UC) who underwent radical nephroureterectomy with bladder cuff excision, reported that preoperative NLR ≥ 3.3 is significantly associated with poorer RFS and CSS in both uni- (both *p* = 0.001) and multivariate analysis (*p* = 0.003 and *p* = 0.017, respectively) [60]. As for inoperable cancers, a retrospective single center study of patients with advanced or metastatic UC reported that elevated NLR values ≥ 3.0 were significantly associated with poorer OS in both uni- (*p* < 0.001) and multivariate analysis (*p* = 0.015) [61]. Advanced/metastatic UC can also respond to immunotherapy. A study on 357 patients with advanced UC unfit for chemotherapy, who were treated with first-line ICIs, reported that NLR > 5.0 was associated with poorer OS in univariate and multivariate analysis (both *p* < 0.001) [62]. Another multicenter retrospective study of 198 mUC patients who underwent pembrolizumab or gemcitabine plus docetaxel second-line treatment reported that low NLR was a significant prognostic factor of OS [63]. However, a retrospective multicenter analysis of 52 mUC patients treated with pembrolizumab as second-line therapy reported no significant differences in CSS between the low- and high-NLR groups, a disparity that may be explained by the small size of the cohort [64].

As for bladder cancer, a large study on patients with non-muscle-invasive bladder cancer who underwent intravesical bacillus Calmette–Guérin therapy after transurethral resection of bladder tumor looked into CRP and NLR as potential prognostic biomarkers for clinical outcomes [65]. While CRP ≥ 0.5 mg/dL was associated with intravesical recurrence, poorer CSS, and bladder cancer progression in uni- and multivariate analysis, NLR ≥ 2.5 was not associated with any of these clinical outcomes [65]. However, NLR seems to be relevant when urothelial bladder cancer is invasive, as revealed by another study where higher NLR was significantly associated with several clinical outcomes in multivariate analysis [66]. In this study, preoperative NLR > 2.8 was associated with a decreased probability of complete response (OR 0.26 [0.14–0.46], *p* < 0.001) and/or partial response (OR 0.36 [0.23–0.56], *p* < 0.001) [66]. Also, pretreatment NLR >2.8 was associated with poorer RFS (HR 1.54 [1.09–2.17], *p* = 0.015), poorer CSS (HR 1.58 [1.06–2.37], *p* = 0.026), and poorer OS (*p* = 0.030). However, postoperative NLR > 2.8 was no longer associated with these survival outcomes [66].

#### 3.2.4. Ovarian, Endometrial, and Testicular Cancer

We found five studies on ovarian cancer, which we discuss in the order of cancer stage and severity. A single-center retrospective study of female patients with stage I epithelial ovarian cancer found that, in univariate but not multivariate analysis, NLR ≥ 3.0 was associated with shorter DFS (HR 22.1 [6.46–75.5], *p* < 0.001) and shorter CSS (HR 56.8 [7.46–433.0], *p* < 0.001) [67]. Another larger study of patients with apparent early-stage epithelial ovarian cancer (stages I–IIIA) who underwent primary surgery reported that preoperative NLR ≥ 3.0 was predictive of recurrence (HR 1.91, [1.02–3.59], *p* = 0.043) and all-cause death (HR 5.80, [1.45–23.26], *p* = 0.013) also in univariate, but not in multivariate analysis [68]. A smaller study of similar patients with stages I–III epithelial ovarian cancer who underwent cytoreductive surgery followed by platinum-based chemotherapy reported differences in NLR between treatment-sensitive (median NLR 2.70) and treatment-resistant (median NLR 1.79) patients [69]. Based on ROC analysis, a cut-off value was established for NLR, with values ≥ 2.56 being prognostic for sensitivity (responsiveness) to platinum-based chemotherapy: 60% sensitivity, 66% specificity, AUC 0.632 [69]. Another study of patients with more advanced epithelial ovarian cancer (stages III–IV), who were treated with chemotherapy alone or in combination with bevacizumab, reported that in the overall cohort, PFS and OS were significantly longer in patients with lower NLR [70]. In multivariate analysis, an NLR ≥ 3.0 was predictive of poorer PFS and OS. However, among patients with high NLR, PFS and OS were better in those treated with chemotherapy plus bevacizumab compared with those treated with chemotherapy alone (*p* = 0.026 and *p* = 0.029, respectively) [70]. This suggests bevacizumab may improve clinical outcomes in patients with high NLR [70]. As for therapy-resistant ovarian cancer, a small study (*n* = 31) reported that high NLR was associated with poorer OS in univariate and multivariate analysis (HR 11.18 [1.10–114.50], *p* = 0.042) when platinum-resistant patients were treated with olaparib plus pegylated liposomal doxorubicin [71].

As for endometrial and testicular cancer, only two studies were included in this review. A small retrospective study on 118 patients with non-endometrioid endometrial cancer treated by surgical resection, reported that NLR ≥ 1.31 predicted DFS in univariate (HR 3.67, [1.45–9.24], *p* = 0.006), but not in multivariate analysis [72]. A similarly sized small retrospective study of 99 patients who underwent orchiectomy for testicular germ cell tumor and were followed up for median 30 months, reported that elevated baseline NLR was significantly correlated with advanced-stage cancer, metastasis, and retroperitoneal lymph node invasion (*p* < 0.05 for all) [73]. Patients were divided into low and high NLR groups (≥3.22), based on the optimum cut-off. A high NLR was modestly predictive for mortality (AUC 0.705, CI 0.541–0.868, 66.7% sensitivity, 73.6% specificity) and cancer progression (AUC 0.625, [0.489–0.760], 50.0% sensitivity, 74.0% specificity) [73].

**Table 5:** The predictive value of neutrophil-to-lymphocyte ratio (NLR) as reported by studies on cancers of the urinary tract and reproductive system.

Cancer Type	Study Details/Treatment	*N*	Median Follow-Up	Outcome	NLR Predictor/Cut-Off	Risk [95% CI]/ Significance	Ref.
mCRPC	Cabazitaxel vs. abiraterone/enzalutamide	255	9.2 mo	OS (poorer)	Per unit increase (baseline)	HR 1.05 [1.02–1.08]	[49]
				rPFS	≥3.38 (baseline)	HR 0.43 [0.27–0.67]	
				rPFS	<3.38 (baseline)	HR 0.69 [0.45–1.06]	
mCRPC (asympt. or minim. sympt.)	Abiraterone acetate vs. placebo	1082	49.2 mo	OS (poorer)	≥2.50 (baseline)	*p* = 0.09	[50]
				OS (better)	<2.50 (baseline)	*p* < 0.0001	
CRPC (M0 or M1)	Pre-/post-docetaxel ARAT agents	303	18.5 mo	rPFS (poorer)	≥2.50 (baseline)	HR 1.53 [1.13–1.89]	[51]
				CSS (poorer)		HR 1.44 [1.04–1.99]	
RCC	Nephrectomy	699	73.0 mo	RFS (poorer)	>3.38	HR 1.68 [1.11–2.51]	[52]
mRCC	TKIs (pazopanib, sunitinib)	150	18.0	OS (poorer)	>2.00	HR 1.27 [0.81–2.00]	[54]
mRCC (or mNSCLC)	Nivolumab monotherapy	161	18.0 mo	OS (poorer)	Increase vs. decrease at week 6 after treatment	HR 2.10	[56]
				PFS (poorer)		HR 2.20	
mRCC	Nivolumab	52	25.2 mo	OS (poorer)	≥3.00 (at 4 weeks)	HR 2.73 [1.08–6.92]	[57]
				PFS (poorer)		HR 2.34 [1.19–4.59]	
Advanced/mRCC	Nivolumab + ipilimumab	35	12.0 mo	PFS (better)	≤4.60	*p* = 0.04	[58]
Advanced RCC	Avelumab + axitinib	434	≥6.0 mo	OS (better)	<2.80	HR 0.51 [0.30–0.87]	[59]
				PFS (better)		HR 0.85 [0.63–1.15]	
	Avelumab + sunitinib	439		OS (better)		HR 0.30 [0.17–0.51]	
				PFS (better)		HR 0.56 [0.41–0.74]	
Localized upper tract UC	Nephroureterectomy with bladder cuff excision	1137	39.1 mo	RFS (poorer)	>3.30	HR 1.38 [1.12–1.69]	[60]
				CSS (poorer)		HR 1.70 [1.10–2.62]	
Advanced/mUC	Inoperable cT4/metastasis	125	12.1 mo	OS (poorer)	≥3.00	HR 1.80 [1.12–2.89]	[61]
Advanced UC	First-line ICI treatment	357	22.0 mo	OS (poorer)	>5.00	HR 1.52 [1.13–2.04]	[62]
mUC	Pembrolizumab/gemcitabine + docetaxel	198	-	OS (better)	Lower	HR 1.97 [1.12–3.45]	[63]
Invasive bladder UC	Neoadjuvant chemotherapy and radical cystectomy	404	49.0 mo	OS (poorer)	>2.80 (pretreatment)	HR 1.47 [1.04–2.09]	[66]
					>2.80 (postoperative)	HR 1.25 [0.88–1.78]	
EOC (stage I–IIIA1)	Primary surgery	359	31.0 mo	Recurrence	≥3.00 (preoperative)	HR 1.18 [0.45–3.06]	[68]
EOC (stage I–III)	Cytoreductive surgery + platinum-based chemotherapy	116	6.0 mo	Treatment responsiveness	≥2.56	*p* = 0.011	[69]
EOC (stage III–IV)	Chemotherapy ± bevacizumab	375	43.0 mo	OS (poorer)	≥3.00	HR 1.44 [1.07–1.93]	[70]
				PFS (poorer)		HR 1.27 [1.00–1.62]	
Non-endometrioid endometrial cancer	Surgical resection	118	41.0 mo	DFS	≥1.31 (preoperative)	HR 1.79 [0.68–4.75]	[72]

Abbreviations: ARAT androgen receptor axis-targeted (agent), CI 95% confidence interval, CSS cancer-specific survival, DFS disease-free survival, EOC epithelial ovarian cancer, HR hazard ratio, mCRPC metastatic castration-resistant prostate cancer, mNSCLC metastatic non-small cell lung cancer, mo months, OS overall survival, (r) PFS (radiographic) progression-free survival, (m) RCC (metastatic) renal cell carcinoma, Ref reference, RFS recurrence-free survival, (m) UC (metastatic) urothelial carcinoma.

### 3.3. Lung Cancer

Lung cancer remains the leading cause of cancer-related deaths among men and women both in the US and worldwide [74]. Non–small cell lung cancer (NSCLC) constitutes around 84% of all lung cancers [74]. Thus, 14 of the 17 studies on lung cancer included in this review focused on the predictive value of NLR in NSCLC. The findings are summarized in Table 6, but some studies are only discussed in the text.

#### 3.3.1. Small-Cell Lung Cancer (SCLC)

Only two studies on SCLC were included in this review. The first one, a large real-world retrospective study of patients diagnosed with SCLC and with an ECOG score of 0–1, reported that NLR > 1.28 was significantly associated with poorer PFS (*p* = 0.002) and OS (*p* = 0.004) [75]. The second study was smaller and included patients with advanced SCLC treated with third-line (or further) anlotinib, but reported similar findings: pretreatment NLR > 3.62 (*p* = 0.025) and elevated post-treatment NLR (*p* = 0.005) were associated with lower rates of response to treatment [68]. Also, post-treatment NLR > 3.62 was predictive for poorer PFS (*p* = 0.006), but not OS (*p* = 0.15) [76].

#### 3.3.2. NSCLC—Mixed or No Treatment

A prospective study of patients with advanced (stage IV) NSCLC at diagnosis reported that NLR ≥ 5.0 was associated with poorer OS in multivariate analysis (*p* = 0.001) and lower one-year survival probability [77]. Another study of 210 NSCLC patients reported that elevated NLR was significantly associated with brain NSCLC metastasis after adjustment for other risk factors (OR 1.20 [1.05–1.37], *p* = 0.006) [78]. Further analysis by subgroups revealed that the association was statistically significant in female, but not male patients (OR 1.52 [1.05–2.20], *p* = 0.026), as well as in patients with the adenocarcinoma pathological type, but not squamous carcinoma (OR 1.30 [1.10–1.54], *p* = 0.002) [78].

#### 3.3.3. NSCLC—Surgical Treatment

A large Japanese retrospective study of 1237 patients with primary NSCLC stages I–III who underwent complete surgical resection reported no prognostic value of pretreatment NLR ≥ 2.56 for DFS or OS (HR and *p*-values not reported) [79]. Very similar results were reported by a smaller study of 254 NSCLC patients who underwent radical surgery, where NLR ≥ 3.18 was only marginally significantly associated with DFS (HR 1.35 [0.95–1.92], *p* = 0.093) and OS (HR 1.40 [0.96–2.06], *p* = 0.077) in univariate analysis, while there was no association in multivariate analysis [80].

#### 3.3.4. NSCLC—Anti-Vascular Tumor Therapy

A prospective study of patients with EGFR-mutant stage IIIB/IV NSCLC who were treated with first-line EGFR-TKIs, reported that NLR ≤ 2.90 was associated with better PFS (*p* = 0.036) and OS (*p* = 0.026) in multivariate analysis [81]. Another study, this time on patients with advanced NSCLC, who were treated with the newly developed multitargeted anti-VEGFR TKI anlotinib, reported similar associations between NLR and clinical outcomes [74]. Pretreatment NLR > 3.41 (*p* = 0.007) and post-treatment NLR increase (*p* = 0.01) were associated with lower disease control rates [82]. Moreover, multivariate analysis revealed that post-treatment NLR increase was associated with poorer PFS and OS (both *p* = 0.001) [82].

One study of 381 patients with advanced non-squamous NSCLC patients reported different NLR-associated outcomes between treatment regimens [83]. Based on the established cut-off, patients were considered as having a low (<3.0) or high (≥3.0) NLR. In the group that received pemetrexed + platinum ± bevacizumab (*n* = 211), low NLR was associated with longer PFS (*p* = 0.026) [83]. Moreover, among a subgroup of 117 patients who were receiving treatment as a first line therapy and who were driver mutation negative, low NLR was further associated with longer OS (*p* = 0.015) [83]. Since no such associations were found in the group of patients treated with paclitaxel + carboplatin + bevacizumab, it was hypothesized that NLR may be a selective prognostic indicator for patients under pemetrexed combination therapy [83].

#### 3.3.5. NSCLC—Immune Checkpoint Inhibitor Therapy

We identified two studies on patients with non-advanced NSCLC treated with ICIs. A multicentric study of NSCLC patients treated with nivolumab as a second (or later)-line treatment reported that a pretreatment NLR ≥ 5.0 was significantly associated with poorer PFS (*p* = 0.028) and OS (*p* = 0.001), but only marginally significantly associated with lower disease control rate (*p* = 0.06) and overall response rate (*p* = 0.16) [84]. Contradicting results were reported by another retrospective study of patients with high PD-L1 expression NSCLC who underwent first-line therapy with pembrolizumab [85]. Here, although NLR ≥ 5.0 showed significant associations with PFS (HR 0.66 [0.45–0.97], *p* = 0.03) and OS (HR 0.57 [0.36–0.89], *p* = 0.01) in univariate analysis, no significant associations were found in multivariate analysis [85]. However, NLR ≥ 5.0 was associated with lower disease control rate (*p* = 0.01) and higher proportions of bone (*p* = 0.01) and liver (*p* = 0.02), but not intracranial (*p* = 0.84), metastases at initial treatment [85].

Regarding advanced or metastatic NSCLC treated with ICIs, a retrospective study of 154 patients with advanced NSCLC, who received second (or further)-line nivolumab, reported that NLR ≥ 4.0 was predictive for shorter PFS (*p* = 0.002) and shorter OS (*p* = 0.04) in multivariate analysis [86]. Similarly, a multicentric study of metastatic NSCLC patients treated with pembrolizumab showed that mean PFS was significantly shorter when pretreatment NLR was > 5.0 (*p* < 0.001) [87]. Furthermore, multivariate analysis revealed that NLR > 5.0 was an independent predictive factor for poorer PFS and OS [87]. However, a smaller prospective study of patients with non-oncogenic driven mNSCLC who were treated with second-line PD-1/PD-L1 inhibitors was not able to detect any significant association between NLR and PFS or OS in multivariate analysis [88].

As for NSCLC patients treated with combined chemotherapy + ICIs, we found two multicenter retrospective studies reporting on different outcomes. The first one, an analysis of patients with extensive-stage NSCLC who were treated with first-line platinum therapy and PD-1/PD-L1 inhibitors, reported that longer PFS (*p* = 0.028), but not OS, was predicted by NLR values ≤ 3.1 in multivariate analysis [89]. The second study, which was larger and included NSCLC patients treated with platinum-based chemotherapy and ICIs, reported that NLR < 3.0 (*p* < 0.01) was an independent predictive factor for immune-related adverse events, which occurred in 15.9% of patients [90].

#### 3.3.6. Malignant Pleural Mesothelioma

We found only one study reporting on this pathological type. This was a prospective study of 184 patients with biopsy-proven malignant pleural mesothelioma, who were followed-up for median 7.5 years. It was concluded that NLR ≥ 4.0 was associated with shorter survival in both univariate and multivariate analysis (HR 0.66 [0.45–0.96], *p* = 0.03) [91].

**Table 6:** The predictive value of neutrophil-to-lymphocyte ratio (NLR) as reported by studies on lung cancer.

Cancer Type	Study Details/Treatment	*N*	Median Follow-Up	Outcome	NLR Predictor/ Cut-Off	Risk [95% CI]/ Significance	Ref.
SCLC	ECOG score 0–1	459	67.6 mo	PFS (poorer)	>1.28	HR 2.01 [1.29–3.14]	[75]
				OS (poorer)		HR 1.70 [1.18–2.46]	
Advanced SCLC	Third (or further)-line anlotinib	53	20.8 mo	PFS (poorer)	>3.62 (after treatment)	HR 2.99 [1.36–6.56]	[76]
				OS		HR 1.70 [0.81–3.54]	
Advanced NSCLC	Newly-diagnosed stage IV	186	8.0 mo	OS	≥5.00	HR 1.91 [1.29–2.84]	[77]
EGFR^pos^ Stage IIIB/IV NSCLC	First-line EGFR-TKI treatment	127	28.1 mo	PFS (better)	≤2.90	HR 0.57 [0.34–0.96]	[81]
				OS (better)		HR 0.49 [0.26–0.92]	
Advanced NSCLC	Third (or further)-line anlotinib	152	7.0 mo	PFS (poorer)	Increase after treatment	HR 1.77 [1.26–2.47]	[82]
				OS (poorer)		HR 2.04 [1.40–2.98]	
Advanced non-squamous NSCLC	PEM + platinum ± Bevacizumab	211	-	PFS (better)	<3.00	HR 0.72 [0.54–0.96]	[83]
	Subgroup: first-line therapy and DM^neg^	117		OS (better)		HR 0.58 [0.38–0.90]	
NSCLC	Second (or further)-line nivolumab	187	-	OS (poorer)	>5.00 (pretreatment)	HR 0.48 [0.29–0.80]	[84]
PD-L1^high^ NSCLC	First-line pembrolizumab	142	15.7 mo	PFS	≥5.00	HR 1.13 [0.71–1.83]	[85]
				OS		HR 0.90 [0.54–1.50]	
Advanced NSCLC	Second (or further)-line nivolumab	154	-	PFS (poorer)	≥4.00	HR 1.91 [1.25–2.92]	[86]
				OS (poorer)		HR 1.69 [1.02–2.82]	
mNSCLC	Second-line pembrolizumab	119	-	PFS (poorer)	>5.00 (pretreatment)	HR 4.47 [2.20–9.07]	[87]
				OS (poorer)		HR 8.09 [2.35–27.81]	
DM^neg^ mNSCLC	Second-line PD-1/PD-L1 inhibitors	66	6.3 mo	PFS	>3.00	HR 1.14 [0.58–2.26]	[88]
ES-SCLC	First-line platinum + PD-1/PD-L1 inhib.	116	-	PFS (better)	≤3.10	HR 0.45 [0.22–0.92]	[89]
NSCLC	Platinum + ICIs	315	-	irAEs	<3.00	OR 2.91 [1.35–6.27]	[90]

Abbreviations: CI confidence interval, DM^neg^ driver mutation negative, ECOG eastern cooperative oncology group, EGFR^pos^ epidermal growth factor receptor activating mutation positive, ES extensive stage, HR hazard ratio, ICI immune checkpoint inhibitor, irAEs immune-related adverse events, mo months, (m)NSCLC (metastatic) non-small cell lung cancer, NLR neutrophil-to-lymphocyte ratio, OS overall survival, PD-(L) 1 programmed death (ligand)-1, PEM pemetrexed, PFS progression-free survival, Ref reference, TKI tyrosine kinase inhibitor.

Given that most studies included in this section focus on NSCLC, the overall conclusion is that NLR appears to be a significant prognostic factor for clinical outcomes in this type of cancer. However, similar to observations in gastric and pancreatic cancers, the prognostic value of NLR seems to be diminished in less extensive, surgically resectable forms of NSCLC.

### 3.4. Breast Cancer

Breast cancer (BC) represents the most prevalent cancer type among women, accounting for 24.5% of all cancer cases in females. In 2020, BC has surpassed lung cancer (11.4%) as the leading cause of global cancer incidence, with a percentage of 11.7% [92]. Considering its profound heterogeneity, BC patients sharing similar prognostic characteristics may experience notably different clinical outcomes, underscoring the imperative for novel prognostic indicators [93]. After searching the literature, nine studies on BC were included in this review.

In a large retrospective study conducted by Jadoon et al. on female patients with BC of all stages, a high NLR > 2.50 was associated with an increase in mortality (*p* = 0.041), yet no association with DFS was observed (*p* = 0.160) [94]. The interpretation of the results on DFS may be limited since the majority of patients were still receiving treatment, leading to a possibly low estimation of true DFS rates [94]. Focusing on locally advanced BC, Eren et al. identified lower NLR < 1.95 as an independent predictor of pathological complete response after neoadjuvant chemotherapy (*p* < 0.001), thus suggesting a decreased treatment response and inevitably worse outcomes in high NLR patients [95]. In a much larger cohort, Muñoz-Montaño et al. also investigated the role of NLR in locally advanced BC treated with neoadjuvant chemotherapy [96]. The study revealed an independent association of high NLR ≥ 2.0 with shorter OS in general (*p* = 0.037), but also in distinct subgroups of patients such those with triple-negative BC (*p* = 0.029) or exhibiting HER2 overexpression (*p* = 0.019) [96]. Compared to most of the other studies in this section, the prognostic value of the NLR was shown here in a large cohort and across different BC phenotypes [96]. Vernieri et al. also investigated the role of NLR in triple negative BC, but in this study, the cohort was smaller, all patients had metastatic BC, and the treatment was platinum-based chemotherapy [97]. By comparing triple negative patients with hormone receptor positive patients, the study revealed decreased OS (*p* < 0.05) with elevated NLR ≥2.5 in both patient groups, suggesting a general prognostic role of the NLR in BC [97]. Interestingly, a difference in survival outcomes was observed between the two groups when treated with carboplatin plus gemcitabine or paclitaxel, high NLR being significantly associated with poor PFS only in triple negative BC patients [97]. Similarly, Nakamoto et al. assessed the association between NLR and survival in advanced triple negative BC in an even smaller cohort undergoing atezolizumab treatment [98]. Here, a low NLR was an independent prognostic marker for longer OS both at baseline and at the start of the second cycle of treatment (*p* < 0.001 and *p* = 0.013, respectively) [98]. Furthermore, lower NLR values at the start of the second cycle of treatment were associated with a longer median time to treatment failure (*p* = 0.002) [98]. Similar to the research conducted by Muñoz-Montaño et al. [88], the study by Sawa et al. addressed various subtypes of metastatic breast cancer (triple negative, HER2+, hormone receptor positive) [99]. Unlike the other mentioned studies, this retrospective study showed no statistically significant association between the NLR and OS [99].

A post hoc analysis conducted by Miyoshi et al. explored the role of the NLR as a predictor of OS in eribulin-treated BC patients, utilizing data from the EMBRACE study [100]. The analysis demonstrated that a decreased NLR < 3.0 was associated with prolonged OS in both eribulin therapy and the physician’s choice of treatment [100]. At the same time, the study showed no statistically significant difference in OS for the high NLR group ≥ 3.0 [100]. Despite the benefits across the treatment groups at a low NLR, no interaction effect was identified between treatment type (eribulin vs. physician’s choice) and baseline NLR values [100]. Since this indicates a consistent association between low NLR and longer OS across different treatment groups, Miyoshi et al. advised utilizing the NLR predominantly as a general prognostic tool instead of a distinct predictive indicator for eribulin therapy [100]. Following the EMBRACE study, which was based on a non-Asian patient population, Takahashi et al. sought to compare these results in a post hoc analysis from an observational study involving Japanese patients with HER2 negative advanced BC under eribulin treatment [101]. Their analysis, based on data from 558 patients, revealed that patients with elevated NLR values ≥ 3.0 had a reduced overall survival OS [101]. Through their study, Takahashi et al. emphasized the applicability of the EMBRACE study’s findings across different ethnicities, aligning with the conclusions of Miyoshi et al. [100] that NLR is a general prognostic marker, rather than a predictor in eribulin-treated BC. With both post hoc analyses referring mainly to the EMBRACE study, the predictive potency of NLR in eribulin-treated patients warrants further exploration, preferably via prospective studies [100,101]. Schettini et al. analyzed the predictive role of the NLR in everolimus-treated hormone receptor positive metastatic BC [102]. Using a higher cut-off value (>4.4) than the other articles on BC, this analysis evaluated three studies, one of which was the BALLET study. Within the BALLET study, which included a total of 2131 patients, 114 patients were eligible for the assessment of the NLR based on the availability of complete blood cell counts. The study revealed a significantly longer PFS in patients with low NLR ≤ 4.4 (*p* = 0.01) [102].

**Table 7:** The predictive value of neutrophil-to-lymphocyte ratio (NLR) as reported by studies on breast cancer.

Cancer Type	Study Details/ Treatment	*N*	Median Follow-Up	Outcome	NLR Predictor/ Cut-Off		Risk [95% CI]/ Significance	Ref.
BC (all stages)	Various treatments	2050	20.0 mo	Mortality (higher)	>2.50		OR 2.08 [1.03–4.19]	[94]
				DFS			*p* = 0.16	
LABC	Neoadjuvant chemotherapy	130	-	pCR (better)	<1.95		OR 3.43 [2.06–5.42]	[95]
LABC	Neoadjuvant chemotherapy	1519	52.9 mo	OS (poorer)	≥2.00		HR 1.40 [1.02–1.95]	[96]
mTNBC, HR+ mBC	Platinum-based chemotherapy	205	-	mTNBC	OS (poorer)	≥2.50	HR 1.96 [1.03–3.71]	[97]
					PFS (poorer)		HR 2.65 [1.36–5.18]	
				HR+ BC	OS (poorer)		*p* = 0.023	
					PFS		*p* = 0.210	
aTNBC	Treatment with atezolizumab	36	-	OS (better)	Lower (baseline)		*p* < 0.001	[98]
					Lower (2nd cycle)		*p* = 0.013	
mBC	Treatment: eribulin vs. physician’s choice	713	-	OS (better)	<3.00		HR 0.75 [0.57–0.99]	[100]
HER2- aBC	Treatment with eribulin	558	-	OS (poorer)	≥3.00		HR 1.55 [1.25–1.92]	[101]
HR+/HER2- mBC	Treatment with everolimus	114	-	PFS (better)	<4.40		*p* = 0.01	[102]

Abbreviations: (a/m) BC (advanced/metastatic) breast cancer, CI confidence interval, DFS disease-free survival, HER2 human epidermal growth factor receptor 2, HR hazard ratio, HR+ hormone receptor positive, LABC locally advanced breast cancer, mo months, OR odds ratio, OS overall survival, pCR pathological complete response, PFS progression-free survival, Ref reference, (a) TNBC (advanced) triple-negative breast cancer.

The use of lower cut-off values for NLR is seemingly a distinct feature of BC studies. Nevertheless, NLR appears to be a broadly useful prognostic marker in BC, showing strong associations with key outcomes such as OS and PFS. Its prognostic value has been validated across several large cohorts, encompassing diverse ethnic populations and various BC phenotypes.

## 4. Discussion

### 4.1. General Considerations

The aim of this review article was to provide clinicians with up-to-date information on the prognostic value of NLR in nonhematologic cancers. Given that our target audience consists primarily of clinicians, many of whom may not be researchers, we chose not to delve into the pathophysiological mechanisms underlying NLR’s prognostic utility. To further enhance the usability of this review, we categorized the cited articles by anatomical location or organ system, enabling clinicians to quickly locate relevant data. This approach was also motivated by the fact that most of the studies we referenced focus on general cancer types rather than offering genetic or molecular characterizations that may have been used for a different classification system. However, where such information was available, we included it. Despite this limitation, we aimed to make the review accessible and easy to navigate for clinicians seeking to update their knowledge on the clinical application of such a widely available biomarker like NLR, without requiring an in-depth understanding of molecular mechanisms.

### 4.2. Synthesis of Evidence

Eighty-eight original research articles were included in this review, covering various types of cancer. The review is organized into four sections (see Figure 1), encompassing a total of 28,050 patients: gastrointestinal cancer (*n* = 8969), cancers of the urinary tract and reproductive system (*n* = 9224), lung cancer (*n* = 4289), and breast cancer (*n* = 5568).

There was considerable variation in the NLR cut-off values used across studies, ranging from below 1.5 to 5.0. Since the majority of studies reported specific NLR cut-off values, we calculated the average NLR cut-off for each major section and for the study as a whole. Recognizing that many studies had small sample sizes while others had large cohorts (*n* = 500–1000 or more), and considering that statistical power is directly related to cohort size, we also calculated a cohort size-weighted NLR (wNLR). The results are as follows: gastrointestinal cancer (average NLR: 3.59; wNLR: 3.59), cancers of the urinary tract and reproductive system (average NLR: 3.00; wNLR: 2.94), lung cancer (average NLR: 3.57; wNLR: 3.09), and breast cancer (average NLR: 2.76; wNLR: 2.50). In summary, the global average NLR cut-off value was 3.35, while the global wNLR cut-off value was 3.08. All of these values, calculated for each section as well as for the study as a whole, approximate the range of 3.0–3.5. This range aligns with the upper reference limit of NLR in a healthy adult population [103,104].

Regarding the predictive role of NLR in cancer, despite the heterogeneity of studies and occasional statistically insignificant or contradictory findings, most studies report significant positive associations between NLR and clinical outcomes, particularly overall survival and progression-free survival. However, certain specific cases should be acknowledged. For instance, in esophageal and rectal cancers, the association between NLR and clinical outcomes appears weak and subtle, which should be interpreted in light of the limited number of studies on these cancers included in this review. Furthermore, as reported in the results section, the association of NLR with clinical outcomes diminishes in less advanced stages of cancer. Specifically, in gastric, pancreatic, and lung cancers, the associations were weak or absent when the cancers were localized and surgery was still a viable therapeutic option. The data suggest that the more advanced the cancer, the stronger the association between NLR and clinical outcomes. This phenomenon may be attributed to the contained immune response and minimal systemic impact of localized cancers.

As for the specificity of NLR, certain studies demonstrate stronger NLR associations within specific treatment cohorts or particular cancer phenotypes. However, the prevailing trend in the literature suggests that NLR is not exclusively indicative of clinical outcomes in specific patient populations, cancer subtypes, or therapeutic responses. Hence, while NLR holds potential for patient stratification and early detection of therapeutic efficacy or failure, its principal utility in the context of cancer lies in its prognostic ability to forecast clinical outcomes across various cancer types.

Our findings are consistent with those of previous reviews and meta-analyses. A 2014 meta-analysis by Templeton et al., which included 100 studies encompassing 40,559 patients, identified a median NLR cut-off value of 4 [7]. Patients with NLR above this threshold exhibited worse outcomes in terms of overall survival (HR 1.81), progression-free survival (HR 1.63), disease-free survival (HR 2.27), and cancer-specific survival (HR 1.61), all with *p*-values < 0.001 [7]. A more recent and extensive umbrella review, published by Cupp et al. in 2020, included 204 meta-analyses from 86 studies [9]. This comprehensive review found that all but one meta-analysis reported a significant hazard ratio greater than 1.00, indicating increased risk. In total, 60 meta-analyses provided strong or highly suggestive evidence for the prognostic value of NLR in cancer [9].

### 4.3. Real-World Clinical Utility of the NLR

This review, alongside previous studies, confirms the prognostic value of the neutrophil-to-lymphocyte ratio (NLR) in cancer patients. However, the question remains: what is the true clinical utility of NLR in everyday oncology practice? Does incorporating NLR into existing predictive models enhance their accuracy and clinical decision-making, or does it represent merely another biomarker with statistical significance but limited practical benefit in patient management?

NLR, either as a standalone marker or in combination with other biomarkers, may serve as a diagnostic tool for cancer and aid in distinguishing between healthy individuals and those with malignancies. This capability has potential clinical utility in guiding diagnostic decision-making. For example, in colorectal cancer, NLR alone demonstrates diagnostic potential, and its accuracy is further enhanced when combined with the platelet-to-lymphocyte ratio (PLR) and carcinoembryonic antigen (CEA) [105,106]. The diagnostic model incorporating all three parameters has proven to be more accurate than any individual marker [105,106]. Similarly, in gastric cancer, NLR has been identified as a diagnostic marker [107], with both NLR and PLR outperforming traditional tumor markers such as CEA and CA19-9 [108]. Moreover, while NLR, PLR, and CEA each hold value in distinguishing between malignant and benign gastric lesions, their combined use yields superior diagnostic accuracy compared to individual markers [109]. In breast cancer, NLR is correlated with TNM staging [94,110] and has diagnostic relevance [110]. However, the combination of NLR, CEA, and CA19-9 performs better in differentiating between breast cancer and fibroadenoma, and thus may be used as a screening and diagnostic tool [110]. In lung cancer, NLR and PLR exhibit moderate discriminatory power between patients and healthy controls, with enhanced accuracy when used together [111]. Additionally, combining NLR with PD-L1 expression has emerged as a promising marker for predicting lung cancer recurrence post-surgery [112,113], particularly in patients with wild-type EGFR [112]. In prostate cancer, NLR can help differentiate between benign and malignant findings prior to needle biopsy, either alone or in conjunction with the free/total PSA ratio [114], and is proposed as a useful tool for reducing unnecessary biopsies [115].

These findings suggest that NLR may contribute not only to prognostication but also to influencing diagnostic processes and therapeutic decision-making. However, the literature presents mixed results, as in most cases the added value of NLR appears to be modest. While its inclusion is certainly advantageous, particularly given that NLR can be easily derived from a CBC, which is a routine test for virtually all hospitalized patients, there is limited evidence to suggest that its prognostic utility translates into clinically significant outcomes, such as improved patient survival through more tailored treatments. This may be partly attributed to the lack of consensus on how the NLR should be implemented in clinical practice and the significant heterogeneity among studies evaluating its clinical utility. As such, expectations regarding the current impact of NLR on improving real-world clinical practice should remain realistic for the time being.

### 4.4. Limitations

This review aimed to provide a comprehensive overview of recent findings on the NLR as a prognostic marker in cancer. Although our findings align with similar previous research, the present review has several limitations. First, despite the rather systematic literature search, this work is not a systematic review and no meta-analysis was performed on the referenced articles. Second, a methodological limitation of this review is that the literature search was conducted exclusively using the PubMed database. Consequently, this review does not purport to encompass all relevant data from the literature between 2018 and February 2024, as there may be pertinent articles that meet the inclusion criteria but are not indexed in PubMed. Third, many individual studies included relatively small cohorts, often comprising fewer than 100–150 patients. And fourth, despite efforts to present the latest advancements, significant variations in research methodology across the studies were observed. This is likely the most significant limitation of the present review, as it restricts both the interpretation and generalizability of the findings. Due to its importance, this issue is specifically addressed in the following section.

### 4.5. The Heterogeneity of NLR Studies

One notable inconsistency across the referenced studies involves the variety of treatments, ranging from surgery, to multiple lines of chemotherapy or various forms of immunotherapy, applied either alone or in combination. Most studies focus on assessing the pre-treatment NLR. However, this approach may not be universally applicable, particularly with treatments involving immune checkpoint inhibitors, which are known to elicit a delayed immune response, indicating that alternative timing for measuring NLR could provide more precise prognostic insights [116,117]. Furthermore, the heterogeneity in pathological types and stages of cancer development reviewed here (from early stage I to advanced or metastatic stages) may have influenced the interpretation of our results. The diversity of cancer subtypes, patient populations, geographical locations, and treatment modalities addressed across the studies complicates the comparison of findings and could affect the generalizability of the results. The clinical outcomes studied also vary widely. Although most studies focus on progression-free survival and overall survival, others assess different clinical endpoints. While the calculation of the NLR is universal, different cut-off values across studies have been used to categorize the NLR as high or low. These cut-off values have been determined by various methodologies such as median value, Youden index, referencing other studies, or arbitrary selection. Differences in follow-up times may have affected findings on survival rates. Additionally, the predominance of small sample sizes and retrospective study designs may present subject to biases and necessitate the need for prospective studies and larger sample sizes to validate our findings.

The heterogeneity among studies may partly be attributed to the numerous factors that influence NLR values. As expected, NLR varies by sex, but the literature presents conflicting findings, with some studies reporting higher values in females, while others show higher values in males [118,119,120,121,122]. Additionally, NLR values differ across ethnic groups, with white Caucasians generally exhibiting higher NLR levels compared to Asians and non-Hispanic Black individuals [118,121]. Lifestyle factors also play a role: smoking and alcohol consumption are associated with elevated NLR, while being married and engaging in regular physical exercise are linked to lower NLR values [121].

However, observations from epidemiological studies involving clinically healthy individuals may not hold relevance in the context of cancer, where the same factors influencing NLR might also affect individual responses to cancer and its treatments. In the context of cancer, both the type and stage of the disease may influence neutrophil involvement. Neutrophils have long been proposed to exhibit either anti-tumorigenic (N1) or pro-tumorigenic (N2) roles within the tumor microenvironment, and emerging evidence supports this theory [123]. Additionally, the localization of tumor-associated neutrophils shifts with cancer progression: they are typically found at the tumor margins in early stages, but infiltrate the tumor mass extensively in later stages, adopting a more pro-tumorigenic role as the disease advances [124]. This shift may help explain our observation that NLR often shows little to no prognostic value in early, localized cancer stages but becomes more significant as the disease progresses.

Beyond cancer type and stage, treatment modalities, which vary considerably across studies, can also impact NLR. A reduction in NLR due to decreased neutrophil counts does not necessarily indicate a better prognosis. For example, neutropenia, the most common complication of chemotherapy [125], may facilitate metastasis [126,127]. Chemotherapy can also paradoxically promote metastasis by activating immune cell-related mechanisms such as neutrophil extracellular traps (which modulate neutrophil phenotype via TGF-β) [128], reactive myelopoiesis [129], and T-cell-dependent regulation of the extracellular matrix [130]. On the other hand, lymphocytes are also affected by cancer therapies as lymphopenia, generally considered unfavorable in cancer [131], is often induced by radiotherapy, chemotherapy, certain immunotherapies, and combination treatments [131,132].

Delving further into the mechanisms underlying NLR variability is beyond the scope of this review. However, it is evident that NLR, which reflects the balance of two blood cell types with key roles in cancer, can fluctuate significantly after cancer onset, particularly following the initiation of treatment. The combination of patient-specific factors, the wide variety of cancer types and subtypes (as characterized by molecular diagnostics), cancer stage, and both current and prior therapies creates a vast array of variables. This complexity often complicates the interpretation of NLR values, even within the same study, let alone across different studies involving diverse cohorts and methodologies. This challenge likely explains why many studies adopt a “default” approach, recording NLR at the time of enrollment without exploring its dynamics throughout the study.

To summarize, as highlighted by both Templeton et al. and Cupp et al., the considerable heterogeneity among studies on this topic poses a significant challenge to drawing definitive conclusions from the literature [7,9]. The absence of robust, standardized data is likely the primary reason why NLR has not gained widespread acceptance, despite substantial evidence linking this marker to clinical outcomes in cancer patients. To facilitate broader adoption of NLR, there is a pressing need for international collaboration aimed at standardizing study methodologies, including adopting a prospective design, setting minimum cohort sizes, and establishing distinct study arms for various cancer types or treatment modalities.

## 5. Conclusions

This review highlights the neutrophil-to-lymphocyte ratio (NLR) as a significant prognostic marker across various types of cancer. NLR is consistently predictive of major clinical outcomes and demonstrates utility especially in advanced disease stages and across multiple treatment modalities, including surgery, chemotherapy, and innovative treatments such as immunotherapy and targeted therapies. In conclusion, we aimed to summarize the major findings of this review by answering six key practical questions.

1. Is NLR a prognostic marker for clinical outcomes in cancer patients?

Yes; data from this review show that NLR can predict clinical outcomes in patients with non-hematologic malignancies.

2 Is NLR a cancer-specific prognosticator of clinical outcomes?

No; NLR exhibits significant fluctuations in response to inflammation, thus being a potentially valuable prognostic marker in various pathologies beyond cancer. When assessing NLR in the context of cancer, careful consideration should be given to excluding concurrent inflammatory conditions.

3 Is the prognostic value of NLR influenced by cancer type and severity?

Unclear, probably yes; limited data from this review suggest that NLR demonstrates attenuated associations with clinical outcomes in certain types of cancer and in cases where the cancer is confined to a localized stage.

4 Is NLR exclusively a prognostic marker in patients undergoing specific therapies?

No; while NLR holds potential for patient stratification and detection of early therapeutic efficacy/failure, it does not appear to be exclusively indicative of clinical outcomes in patients undergoing specific therapies.

5 What is an appropriate NLR cut-off value for use in cancer studies?

NLR cut-off values in cancer studies vary widely in the literature, with the optimal cut-off potentially depending on the type of cancer and study design. While it is advisable to determine an NLR cut-off using statistical tools, the results should be interpreted in the specific clinical context of the study. If an indicative NLR cut-off is required, a value in the range of 3.0–3.5 may be considered.

6 Does NLR have clear indications for use in oncology practice?

Not yet; although NLR shows promise as a biomarker from a statistical standpoint, its contribution to current diagnostic processes and predictive clinical models remains limited. Furthermore, the variability in study methodologies complicates the ability to draw broadly applicable conclusions, making it challenging to establish concrete clinical indications for its use in oncology practice at this time.
